# Risk Factors of Allergic Disease: A Study with a Large Data Set

**DOI:** 10.1155/2017/8969352

**Published:** 2017-10-10

**Authors:** In-Hwan Oh, Yeong-Ho Rha, Takao Fujisawa, Kyung Suk Lee

**Affiliations:** ^1^Department of Preventive Medicine, College of Medicine, Kyung Hee University, Seoul, Republic of Korea; ^2^Department of Pediatrics, School of Medicine, Kyung Hee University, Seoul, Republic of Korea; ^3^Institute for Clinical Research, Mie National Hospital, Mie, Japan; ^4^Department of Pediatrics, CHA Bundang Medical Center, CHA University School of Medicine, Seongnam, Republic of Korea

Allergic diseases (e.g., asthma, allergic rhinitis, and atopic dermatitis) are a major public health problem, and the prevalence among children is increasing worldwide [1, 2]. Many studies have revealed the cause, pathogenesis, prevalence, and risk factors for allergic diseases for decades. However, we do not know all fields of allergic diseases and we have still explored the risk factors for allergic diseases.

Nowadays, large amounts of data (big data) are digitally stored and have been successfully used in many fields such as science, economics, and politics [3]. The interest and application of big data in medical science is also growing because big data have several advantages that expand the capacity to generate and disseminate new knowledge [3]. The sources of big data intended for medical application include government agencies, medical organizations, and other credible health-related institutions [3–5]. Regarding allergic diseases, big data is useful in investigating the causes, epidemiology, genetics, treatment, and economic burden [4–6].

In this special issue, six studies were published regarding the risk factors of allergic diseases.

W. S. Lee et al. analyzed the relationship between home remodeling, food allergy, and atopic dermatitis among children in Seongnam, Korea. In this large population study, home remodeling (odds ratio = 3.40) and food allergy (odds ratio = 3.95) are the risk factors of atopic dermatitis. K. S. Lee et al. also found that age, smoking, and elevated total IgE levels were other risk factors for atopic dermatitis in Korean children and adolescents by using the 2010 Korea National Health and Nutrition Examination Survey (KNHANES) which comprised a large sample. Y. Feng et al. investigated the prevalence and features of ocular allergy and comorbidities among school children in Shanghai, China, by using a questionnaire. They found a 28% prevalence of symptoms of ocular allergy. S.-J. Yi et al. investigated the risk for atopic eczema among children living in areas surrounded by large and busy roads in Seoul, Korea. They found that the onset of atopic eczema correlated with road density (odds ratio: 1.08) and proximity (odds ratio: 1.15) but not asthma and rhinitis. The study by S.-M. Koo et al. is the only one that focused on the adult population in this special issue. They analyzed the trend of use of asthma medication among asthmatic pregnant women using the Health Insurance Review and Assessment Service (HIRA) database of Korea. They found that despite the low adherence to asthma medication, exacerbations were less frequent during pregnancy. S. H. Choi et al. investigated the repeatability and safety of measuring lung function using impulse oscillation systems during bronchoprovocation testing of preschool children.

Most of the studies in this special issue focused on the relationship between environmental risk factors and allergic diseases in children and found or confirmed the relationship using large data sets. These results proved that finding the risk factors for allergic diseases using big data is promising.

This special issue is just the beginning of big data research in allergic diseases. We hope that readers will be interested in allergic diseases and big data and that this special issue could help them to devote their time to research.

*In-Hwan Oh* *Yeong-Ho Rha* *Takao Fujisawa* *Kyung Suk Lee*
